# Coronavirus Infection in Neonates: Neurodevelopmental Outcomes at 18 Months of Age

**DOI:** 10.1155/2023/6140085

**Published:** 2023-01-02

**Authors:** Mariam Ayed, Zainab Alsaffar, Zainab Bahzad, Yasmeen Buhamad, Ali Abdulkareem, Alaa AlQattan, Alia Embaireeg, Mais Kartam, Hessa Alkandari

**Affiliations:** ^1^Neonatal Department, Maternity Hospital, Kuwait City 900015, Kuwait; ^2^Pediatric Department, Farwaniya Hospital, Kuwait City 81400, Kuwait; ^3^Population Health Department, Dasman Diabetes Institute, Kuwait City, Kuwait

## Abstract

**Background:**

Although most neonates with severe acute respiratory syndrome coronavirus-2 (SARS-CoV-2) infection experience only mild disease, its impact on neurodevelopmental outcomes is unknown. This study aimed to assess the 18-month neurodevelopmental outcomes of infants who had SARS-CoV-2 infection as neonates.

**Methods:**

The authors conducted a prospective cohort study of neonates diagnosed with SARS-CoV-2 infection from June 2020 to December 2020 through nasopharyngeal coronavirus disease 2019 (COVID-19). A total of 58 neonates were identified from the Kuwait National COVID-19 Registry and enrolled. Historical controls were selected from the neonatal follow-up registry and matched in a 2 : 1 ratio based on sex and gestational age. When the subjects were 18 months of age, their neurodevelopmental outcomes were assessed by two trained assessors using the Bayley Scales of Infant and Toddler Development-3rd Edition (BSID-III).

**Results:**

Forty children diagnosed with SARS-CoV-2 infection were included in the final analysis. The median age at infection was 18 days (range: 10–26 days). Eighteen (45%) patients were asymptomatic, 15 (37.5%) had a sepsis-like presentation, 5 (12.5%) exhibited respiratory distress, and 2 (5%) had a multisystem inflammatory syndrome in children (MIS-C)-like presentation. At the 18 months follow-up, only one child showed a severe developmental delay and one child had a language delay. BSID-III outcomes did not differ significantly between the SARS-CoV-2-infected and control groups.

**Conclusions:**

There was no difference in neurodevelopmental outcomes at 18 months in children infected with SARS-CoV-2 compared with controls, although longer neurodevelopmental follow-up studies are required.

## 1. Introduction

Severe acute respiratory syndrome coronavirus-2 (SARS-CoV-2) infection in children has been associated with a multisystem inflammatory syndrome and coronavirus disease 2019 (COVID-19), which are recognized as two distinct clinical entities [1–3]. It is widely known that a smaller proportion of children than adults become ill when infected with SARS-CoV-2 [4].

Neurological symptoms have previously been identified in pediatric cases of COVID-19 [5], with approximately 22% of hospitalized children being affected by neurological manifestations, such as smell/taste disorder, headache, and stroke [6]. Intracranial hypertension [7] and acute encephalitis [8] have also been reported in children with multisystem inflammatory syndromes.

There are several potential mechanisms by which CNS involvement may occur in association with COVID-19. Animal studies on SARS-CoV-2 have suggested that angiotensin-converting enzyme 2, acting as the viral receptor, may mediate coronavirus-related neuronal damage, and there is also evidence to suggest that the virus can infect the cerebrovascular endothelium and brain parenchyma, particularly in the medial temporal lobe, resulting in apoptosis and necrosis [9]. In addition, postmortem studies of the human brain have yielded evidence indicating that human coronavirus variants and SARS-CoV can infect both neurons and glia, and increased cytokine serum levels associated with SARS-CoV infection may indicate increased cytokine production [9]. Antineuronal and antiglial autoantibodies have been detected in the cerebrospinal fluid of patients with COVID-19 and neurological symptoms, which raises the possibility that autoimmune mechanisms are involved in the pathogenesis of COVID-19 with neurological involvement [10].

Although most neonates with SARS-CoV-2 infection have mild disease, the impact on long-term neurodevelopmental outcomes is currently unknown. It has been shown that birth during the COVID-19 pandemic, but not in utero exposure to maternal SARS-CoV-2, is associated with differences in neurodevelopmental outcomes at 6 months of age [11]. Another study showed that newborns exposed in utero to SARS-CoV-2 exhibit largely normal neurological development in the first few months of life [12]. However, these studies provided only a short-term perspective on neurodevelopmental outcomes.

The objective of this study was thus to assess the neurodevelopmental outcomes at 18 months in infants who had confirmed SARS-CoV-2 infection as neonates. It has been hypothesized that neonatal SARS-CoV-2 infection may lead to adverse neurodevelopmental outcomes.

## 2. Materials and Methods

### 2.1. Patient Enrollment

We conducted a prospective cohort study of neonates diagnosed with SARS-CoV-2 infection between June 2020 and December 2020. Neonates underwent nasopharyngeal COVID-19 PCR according to our national COVID-19 protocol. Neonates were confirmed for testing if they fulfilled one of the following criteria: (1) contact with a known COVID-19 patient; (2) symptoms of fever, cough, or respiratory distress; or (3) tested as a screening test prior to hospital admission (per our national guideline to screen all patients before hospitalization). Verbal informed consent was obtained from the children's parents before conducting the study. Ethics approval was granted by the Ministry of Health of Kuwait (approval numbers: 2021–1638).

A total of 58 neonates were identified from the Kuwait National COVID-19 Registry and enrolled in the prospective SARS-CoV-2 infection outcome cohort. Of these, 6 were lost to follow-up, 2 refused to participate in the neurodevelopmental outcome study, 6 left the country, and 1 died from COVID-19 complications. Four neonates were excluded as we were unable to determine their COVID-19 status due to equivocal results; all neonates were asymptomatic and were tested due to positive contact with COVID-19 patients; therefore, repeat testing was not done. Historical controls were selected from the neonatal follow-up registry and matched in a 2 : 1 ratio based on sex and gestational age.

Data were retrieved from hospital records and included demographic details, parental education, maternal age at the birth of the child, clinical features, hospital course, and morbidity assessment, including any seizure episode or rehospitalization.

### 2.2. Definitions

Respiratory distress was defined as the presence of either increased work of breathing or oximetry of <93%. We used the multisystem inflammatory syndrome (MIS-C) definition of the CDC and AAP [13, 14]. MIS-C often occurs in patients aged <21 years, with the following clinical criteria: a minimum 24-hour history of subjective or objective pyrexia ≥38.0°C, severe illness requiring hospitalization, and involvement of two or more organ systems (i.e., cardiac, renal, respiratory, hematologic, gastrointestinal, dermatologic, or neurological). Laboratory evidence of inflammation was defined as one or more of the following: elevated C-reactive protein (CRP), erythrocyte sedimentation rate, fibrinogen, procalcitonin, D-dimer, ferritin, lactate dehydrogenase, or interleukin-6; elevated neutrophils or reduced lymphocytes; and low albumin.

A diagnosis of central nervous system comorbidities and chromosomal abnormalities was ruled out in the enrolled neonates.

### 2.3. Assessment of Neurodevelopmental Outcomes

When the subjects were 18 months of age, their neurodevelopmental outcomes were assessed by two trained assessors using the Bayley Scales of Infant and Toddler Development-3rd Edition (BSID-III) [15]. These scales are age-standardized, with composite cognitive, motor, and language scales with a normative mean of 100 and a standard deviation (SD) of 15. A diagnosis of moderate developmental delay was given for the worst composite score of 70–84 in one or more of the three domains. However, the severe developmental delay was used for a score of <70 for any of the three domains or when a score could not be assigned due to severe mental deficiency or cerebral palsy, as appraised using the gross motor function classification system (GMFCS) [16]. Additionally, both blindness and deafness were evaluated by a pediatrician. Blindness was defined as a visual acuity of less than 6/60 in the better eye, and deafness was defined as a hearing impairment requiring amplification, cochlear implant, or worse [17]. We also obtained anthropometric variables, including head circumference, length, and weight, measured using standard techniques and growth standards of the World Health Organization.

### 2.4. Statistical Analysis

Categorical data are presented as proportions and continuous data as the median and interquartile range (IQR). Differences in clinical data between infants were compared using Pearson's chi-square test for categorical variables (Fisher's exact test was used when a frequency was less than 5) and with the Wilcoxon rank-sum test for continuous variables. The statistical significance was set at *P* < 0.05. All statistical analyses were performed using Stata/IC 14.2 (StataCorp, College Station, Texas, 2015).

## 3. Results

Forty children were diagnosed with SARS-CoV-2 infection and included in the final analysis. The demographic details of the study cohort are presented in Table 1. The median age at infection was 18 days (IQR: 10–26 days). Two of the infected neonates were born to mothers who were SARS-CoV-2 infected at the time of birth. Eighteen (45%) patients were asymptomatic; 17 were tested due to contact with known COVID-19 patients, and 1 was tested before inguinal hernia surgical repair. Fifteen (37.5%) had sepsis-like presentations (fever, lethargy, and poor feeding), five (12.5%) had respiratory distress, and two (5%) had MIS-C-like presentations. Of the 5 patients with respiratory distress, 4 received supplemental oxygen, and 1 received a humidified high-flow nasal cannula. Only one patient with the MIS-C-like presentation required invasive respiratory and circulatory support. None of the infants with MIS-C had cardiac arrhythmia or dilated coronaries. None of the symptomatic infants had a concomitant positive viral or bacterial screening. The median duration of hospital stay was 4 days (IQR: 2–14 days).

During the 18-monthfollow-up period, two infants required rehospitalization: one with a simple febrile seizure at 7 months of age and the second with acute bronchiolitis at 4 months of age.

The maternal and infant characteristics did not significantly differ between the exposed and unexposed groups (Table 2).

At 18 months, there were no differences in the Bayley-III composite scores between the exposed and control groups. In the SARS-CoV-2-exposed group, one infant had a score of less than 70 in the motor, cognitive, and language composite scores, and one infant had a language composite score of less than 85. The infant with a score <70 in all domains had a neonatal MIS-C-like presentation. She presented with lethargy, fever, tachycardia, and hypotension at the age of 8 days and required invasive ventilation, inotropic support, and intravenous immunoglobulin therapy. She developed seizures on day 10 and had markedly increased C-reactive protein (CRP), myocardial enzyme, and D-dimer levels. A lumbar puncture was not performed because of instability. Her septic workup, including blood and endotracheal cultures, was negative. Magnetic resonance imaging (MRI) revealed right temporal and bilateral basal ganglia hemorrhagic foci with abnormal cortical and subcortical signal intensity on T2-weighted images in both the frontoparietal and posterior parietal regions. The second child, who scored less than 85 in the language composite score, presented with fever and pneumonia, which were confirmed by chest radiographic imaging at 14 days of age. He received supportive treatment for 5 days, including a humidified high-flow nasal cannula for 3 days. He had normal hearing and a normal neurological examination.

## 4. Discussion

This prospective cohort study compared the neurodevelopmental outcomes at 18 months of age in 40 children diagnosed with SARS-CoV-2 infection with a group of 80 historical controls matched in terms of maternal and infant characteristics at baseline. The study found no significant difference in Bayley-III composite scores between the two groups, indicating similar neurodevelopmental outcomes.

Overall, the prognosis of neonates with SARS-CoV-2 infection was good, with only one infant having a severe developmental delay and one having a language delay. Almost half of our patients were asymptomatic, and one with an MIS-C-like presentation developed seizures and a brain injury on MRI. There has been significant interest in the manifestations of SARS-CoV-2 infection in the pediatric population, particularly neurological manifestations [19, 18]. The systemic inflammatory response commonly observed in adults is much rarer in the pediatric population [19]. However, long COVID may well be an under-recognized phenomenon [20]. The challenge of recognizing the clinical characteristics of COVID-19 among other neonatal diseases is apparent [21]; some recognized manifestations of SARS-CoV-2 infection in children include stroke [22], seizures, seizure-like activities [23–25], headaches [23], dizziness [23], meningoencephalitis [23, 26–28], and opsoclonus [29]. Rare neurological complications include intracranial hemorrhage, cranial neuropathies, Guillain–Barré syndrome, and visual impairments [30]. Many neurological manifestations have been observed in association with MIS-C [31]. The underlying pathophysiology driving these clinical manifestations of SARS-CoV-2 infection remains to be fully elucidated, although immune dysregulation and autoreactivity have been shown to correlate with disease severity in children [32], and the use of blood biomarkers for the diagnosis of brain injury in patients with COVID-19 has been proposed [33]. Neuroimaging performed in children with SARS-CoV-2 infection most commonly reveals postinfectious immune-mediated acute disseminated encephalomyelitis-like changes in the brain, myelitis, and neural enhancement [34]. Focal cerebral arteriopathy [35] has also been identified. Diffusion restriction [36] and cerebral white matter injury [37] have also been described in the neonatal population.

The neurodevelopmental outcomes of neonates infected with SARS-CoV-2 are of particular interest, given the potential implications for the child's long-term prognosis and functional outcomes. The effect of SARS-CoV-2 infection, specifically in neonates, has been relatively understudied [38, 39]. There is evidence that most infected neonates are asymptomatic or experience only mild disease [38, 40], although risks of direct and indirect adverse health outcomes have been identified [39, 41]. In this study, only one infant had a severe developmental delay and one had a language delay at 18 months. It is difficult at this stage to establish a direct link between SARS-CoV-2 infection and adverse neurodevelopmental outcomes. However, we can speculate that, like with any other viral infection, systemic inflammation induced by cytokines can lead to neuronal injury [42]. Even without meningitis, the infection can lead to brain injury and neurodevelopmental impairment [42].

Few studies have investigated neurodevelopmental outcomes in neonates exposed to SARS-CoV-2 infection [43]. However, one study showed that in utero exposure to maternal SARS-CoV-2 infection is not associated with adverse neurodevelopmental outcomes at 6 months of age [11]. Another study showed that newborns exposed to SARS-CoV-2 in utero have largely normal neurological development in the first few months of life. A multicenter observational study is also underway with the aim of reviewing neurodevelopmental outcomes at 6 months [44]. However, long-termfollow-up is required to provide a more accurate picture. Telehealth may be a useful method for monitoring the long-term neurodevelopmental outcomes of neonates who develop SARS-CoV-2 infection [45].

This study has several limitations. First, the control population was a historical cohort; therefore, their data may be incomplete. Second, the sample size is relatively small. Third, we were unable to control for unmeasured confounders in this study. The disease severity could confound the relationship between adverse neurodevelopmental outcomes and COVID-19 disease in our infants. We also excluded infants with equivocal PCR testing; this exclusion could lead to selection bias.

## 5. Conclusion

This study demonstrated no difference in neurodevelopmental outcomes in children infected with SARS-CoV-2 compared with a historical control group. However, additional long-termfollow-up is required to ensure this remains true after 18 months. Furthermore, neurodevelopmental outcomes are likely to be largely normal, as most neonates are asymptomatic or have mild symptoms. Neonates with severe COVID-19 or neurological involvement are likely at a higher risk of neurodevelopmental impairment.

## Figures and Tables

**Table 1:** Demographic characteristics of the study subjects.

Variable	SARS-CoV-2 exposed, *N* = 40	Historical control, *N* = 80	*P* value
Gestational age, week	38 (37–40)	39.2 (38–40)	0.716
Sex			0.872
Male	19 (47.5%)	39 (48.8%)	
Female	21 (52.5%)	41 (51.2%)	
Birth weight, grams	3000 (2500–3420)	3457 (3147–3765)	0.12
Mode of delivery			0.532
Caesarean section	13 (57.5%)	42 (52.5%)	
Vaginal birth	17 (42.5%)	38 (47.5%)	
5 minutes Apgar score	8 (7–10)	8 (7–9)	0.128
Maternal education			0.104
Educated	1 (2.5%)	5 (6.2%)	
Elementary education	2 (5%)	2 (4.5%)	
High school	4 (10%)	7 (68.7%)	
Diploma	13 (32.5%)	29 (36%)	
Bachelor/PhD	20 (50%)	37 (46.2%)	
Maternal age, years	27.3 (25–32)	27.7 (26–31.7)	0.296
Paternal education			0.109
Educated	1 (2.5%)	0	
Elementary education	5 (12.5%)	10 (12.5%)	
High school	4 (10%)	12 (15%)	
Diploma	12 (30%)	28 (35%)	
Bachelor/PhD	18 (45%)	30 (37.5%)	
Age at follow-up, months	18.6 (18.2–19.5)	18.7 (18.4–19.3)	0.174

Data are expressed as number (%) and median (interquartile range).

**Table 2:** Follow-up outcomes at 18 months.

Variables	SARS-CoV-2-infected group, *N* = 40	Control group, *N* = 80	*P* value
Bayley-III			
Motor composite score	100 (91–107)	97 (88–107)	0.101
71–85	0	2 (2.5%)	0.786
<70	1 (2.5%)	1 (1.2%)	

Fine motor	11 (10–12)	10 (9–12)	0.21
Gross motor	9 (7–10)	9 (6–10)	0.231

Cognitive composite score	105 (100–115)	110 (100–115)	0.952
71–85	0	3 (3.7%)	0.261
<70	1 (2.5%)	0	

Language composite score	100 (89–109)	100 (89–109)	0.785
Expressive	9 (7–12)	9 (8–11)	0.971
Receptive	10 (8–12)	10 (8–12)	0.645
71–85	1 (2.5%)	3 (3.7%)	0.864
<70	1 (2.5%)	1 (1.2%)	

Cerebral palsy	1 (2.5%)	2 (2.5%)	0.131
Seizure	1 (2.5%)	0	1
Deafness	0	1 (1.2%)	0.293
Blindness	0	0	
Weight (kg)	11.1 (10.2–12.7)	11.7 (10.7–12.9)	0.212
Length (cm)	82 (79–84)	83 (78–86)	0.304
Head circumference (cm)	47.6 (46.9–48.8)	48.5 (47.5–49)	0.138

Data are expressed as number (%) or median, interquartile range (IQR).

## Data Availability

The analysis code compiled using Stata software is available from the corresponding author. Data sharing with readers is not available because of the requirements of the Ethics Committee of the Ministry of Health of Kuwait.

## Abbreviations

BSID-III: Bayley Scales of Infant and Toddler Development-3rd Edition
COVID-19: Coronavirus disease 2019
GMFCS: Gross motor function classification system
IQR: Interquartile range
MIS-C: Multisystem inflammatory syndrome in children
SARS-CoV-2: Severe acute respiratory syndrome coronavirus-2
SD: Standard deviation.
